# Macro-, meso- and microstructural characterization of metallic lattice structures manufactured by additive manufacturing assisted investment casting

**DOI:** 10.1038/s41598-021-84524-y

**Published:** 2021-03-02

**Authors:** V. H. Carneiro, S. D. Rawson, H . Puga, P. J. Withers

**Affiliations:** 1 CMEMS-UMinho, University of Minho, Campus of Azurém, 4800-058 Guimarães, Portugal; 2 Department of Materials, The Henry Royce Institute, The University of Manchester, Manchester, M13 9PL UK

**Keywords:** Mechanical engineering, Engineering, Materials science

## Abstract

Cellular materials are recognized for their high specific mechanical properties, making them desirable in ultra-lightweight applications. Periodic lattices have tunable properties and may be manufactured by metallic additive manufacturing (AM) techniques. However, AM can lead to issues with un-melted powder, macro/micro porosity, dimensional control and heterogeneous microstructures. This study overcomes these problems through a novel technique, combining additive manufacturing and investment casting to produce detailed investment cast lattice structures. Fused filament fabrication is used to fabricate a pattern used as the mold for the investment casting of aluminium A356 alloy into high-conformity thin-ribbed (~ 0.6 mm thickness) scaffolds. X-ray micro-computed tomography (CT) is used to characterize macro- and meso-scale defects. Optical and scanning electron (SEM) microscopies are used to characterize the microstructure of the cast structures. Slight dimensional (macroscale) variations originate from the 3D printing of the pattern. At the mesoscale, the casting process introduces very fine (~ 3 µm) porosity, along with small numbers of (~ 25 µm) gas entrapment defects in the horizontal struts. At a microstructural level, both the (~ 70 μm) globular/dendritic grains and secondary phases show no significant variations across the lattices. This method is a promising alternative means for producing highly detailed non-stochastic metallic cellular lattices and offers scope for further improvement through refinement of filament fabrication.

## Introduction

Cellular lattices are characterized by an interconnected solid scaffold configuration, showing high specific strength and very low relative density^1,2^. As a result, this class of materials is being considered for a wide range of potential applications across transportation (e.g. aerospace/aeronautic^3–6^, railway^7–9^, etc.), medical (e.g. scaffolding/stenting^10–12^), among other industries^13–15^. In applications that demand significant load-bearing capability, metallic lattice structures are most appropriate.

Stochastic cellular lattices are classically produced by the introduction of foaming/blowing agents in alloy melts^16–18^, although they may also be produced by powder metallurgy^19–21^, wire-weaving^22–24^, additive manufacturing^25–28^ and casting^29–32^.

Regular periodic lattice structures have the advantage that the properties can be more precisely tailored. Additive manufacturing techniques are reported to be able to produce such lattices with thin-walls and struts with thicknesses down to ~ 0.24mm^33–35^, however, they tend to have issues in terms of poor fine detail/dimensional control. Further, at a finer scale metal-based additive manufacturing techniques have been reported to promote meso- and micro-scale defects, such as: porosity, delamination, and un-melted powders, as well as highly anisotropic grain morphologies as a function of build direction^35–44^.

Some of these issues may be addressed by metal casting techniques, where there have been recent advances on the filling of thin-walled molds^45^ allowing good dimensional control^46–50^ coupled with good microstructural control^51–53^. However, the main difficulties associated with casting techniques are frequently related to mold production^54^. To address this issue, recent research has focused on developing novel hybrid additive manufacturing assisted investment casting techniques^55–57^ which is the focus of the current study.

Here we take a look at the metallurgical aspects of A356 aluminium alloy lattice structures. A356 is the most common Al alloy used to cast components for transportation industries due to its capability to be cast in ceramic block to produce thin-walled and complex geometries^58^. As a hypoeutectic aluminium alloy, the microstructural characterization of A356 is often concerned with the size and shape of the α-Al matrix^58–61^, eutectic Si^62–65^, Mg_2_Si and Fe-based intermetallic compounds^66–69^, such as Chinese script-shaped α-Al8Fe2Si and needle-shaped β-Al_5_Fe_2_Si. In as-cast samples, previous to solution treatment, Mg_2_Si are precipitated in the grain boundaries^70,71^ and, due to the presence of Mg, Chinese script-shaped π-Al_8_FeMg_3_Si_6_ may also be observed^48,49^.

We characterize hybrid additive manufacturing assisted investment cast lattice structures at the macro-, meso- and microstructural scales using a combination of X-ray micro-computed tomography (μCT) and optical/electron microscopy in order to assess its promise for the manufacture of fine periodic lattice structures. While the manufacturing approach is considered to be applicable to many lattice design, here the selected lattice topology is a classic reentrant honeycomb configuration which has been widely explored for its auxetic (i.e. negative Poisson’s ratio) behavior. While having a bending dominated deformation mechanism, it has also been shown to possess high specific strength, relatively to other more common open cellular lattices.

## Results and discussion

### Macrostructural characterization: dimensional changes

**Figure 1:**
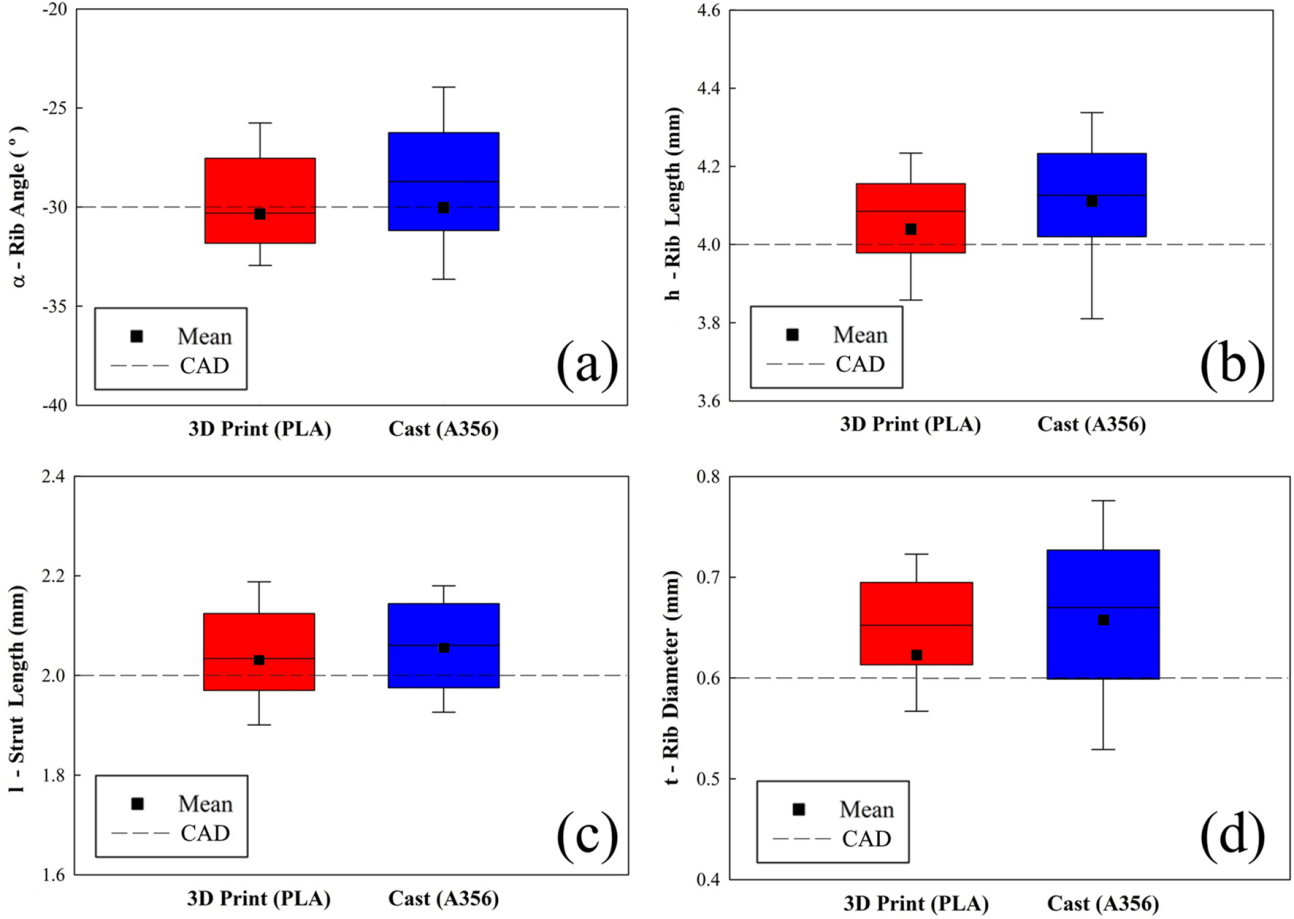
Box plots of dimensional analysis by X-ray μCT (n = 50, dashed line is the theoretical CAD dimension) of the 3D printed pattern and final Al casting with reference to the original CAD design for (**a**) rib angle, (**b**) rib length, (**c**) strut length and (**d**) rib diameter.

Figure 1 summarizes the non-conformance variation in the dimensions of the sacrificial Polylactic Acid (PLA) pattern and the final A356 lattice structures obtained by 3D X-ray μCT relative to the CAD model design. As must be the case, given the castings derive from the molds made from the 3D printed patterns, the highest deviations are observed in the final as-cast A356 samples, however it is also clear that most of the deviation is transferred over from those introduced during fabrication of the pattern.

Regarding the deviations introduced during the production of the PLA pattern (Step I), these can be primarily attributed to the precision of the 3D-printer stepper motor (resolution ± 12.5 μm). Additionally, vertical struts may become bent due to interactions with the rigid nozzle, whereby molten filament in the nozzle may adhere to already deposited PLA as well as due to thermal expansion/contraction effects due to temperature mismatch and oscillations between glass/rubbery states^72–74^.

Deviations between the PLA pattern and the final A356 lattice structure (Step II) can be attributed to dimensional changes that occur during two distinct processing stages; (i) forming the investment cast: due to the expansion of the investment gypsum mold during its curing which involves the loss of water and the transformation of gypsum from α (cristobalite) to β (quartz)^75^; and (ii) during/after A356 melt casting: due the contraction of the A356 alloy during the solidification and subsequent cooling^76,77^. The net result is that the final A356 cast samples tend to display slightly larger dimensions than the PLA samples.

The data in Fig. 1 was also used to perform a statistical analysis (one-way ANOVA on Ranks with Kruskal–Wallis test—for significance of p < 0.05) to determine the origin of dimensional deviations in the manufacturing process. This allows the comparison of the dimensional divergence in (i) Step 1 between the CAD model and the sacrificial 3D-printed PLA pattern; and (ii) Step 2—between PLA pattern and the final A356 lattice. According to the results in Table 1, it is evident that the angular dimensions (α, rib/strut angle) are preserved through the manufacturing process showing no statistical difference between CAD model, pattern and final casting; the same is not true for linear dimensions. It is apparent that strut length (l) and rib/strut thickness (t) show statistically significant differences in the first manufacturing step. Thus, it may be concluded that the fundamental sources of dimensional variation in the manufacturing process originate from the 3D printing of the pattern.

**Table 1:**
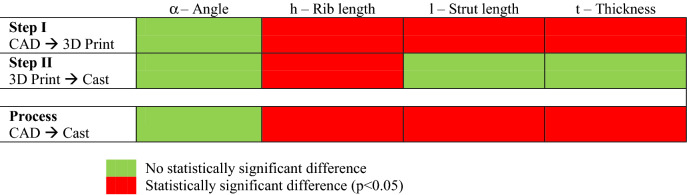
Statistical significance of the deviation of the strut dimensions during the two steps of the manufacturing process.

The analysis of both the deviations from the CAD model and their statistical distribution upon making the PLA pattern (Stage I) and then casting the A356 lattice (Stage II) are shown in Fig. 1 and Table 1 This shows that the final cast samples display only small variations relative to the CAD model (0.1º and 0.06–0.12 mm). In statistical terms, however, it is shown that deviation in the linear dimensions in the final cast samples relative to the CAD model do not follow a normal distribution and are statistically significant.

### Mesoscale characterization—details and defects

**Figure 2:**
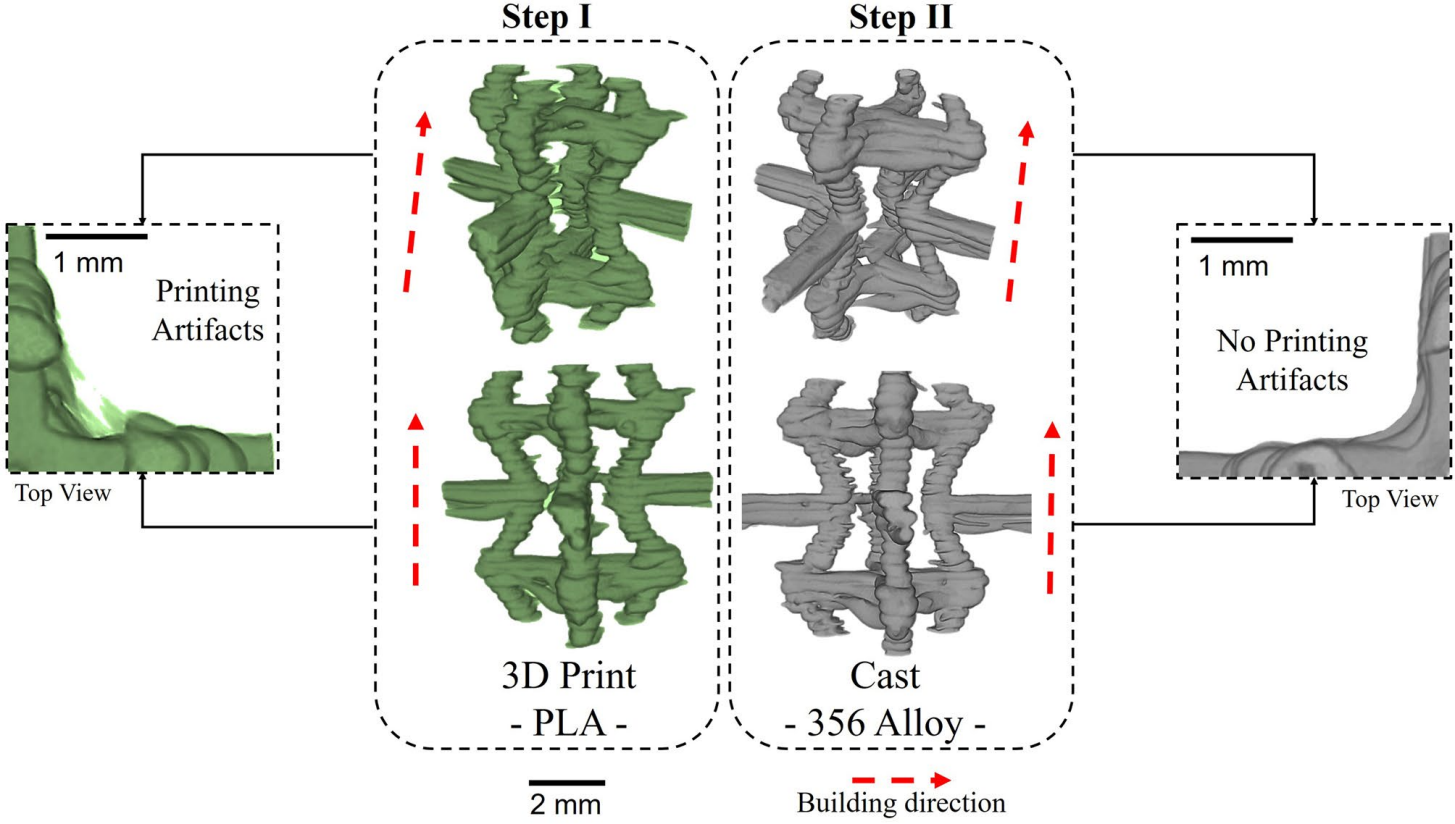
Regions of interest from the µCT renderings (Avizo Standard segmentation) for the periodic cell during the two steps in the manufacturing process.

X-ray μCT was also used to characterize the details and defects. Figure 2 confirms the conclusion from the previous section in that the layers deposited by the fused filament fabrication (FFF) process are faithfully transferred to the final samples such that most of the mesoscale defects can be traced to stage I (from CAD to 3D-printed PLA pattern). It is also noteworthy that the inability to immediately stop filament extrusion during the printing process and the coalescence/viscoelasticity of the PLA^78^ mean that fine artifacts are introduced into the PLA pattern. However, it is evident that these artifacts are not present in the final A356 sample, probably because they are extremely thin such that the melt is not able to fill their cavities.

**Figure 3:**
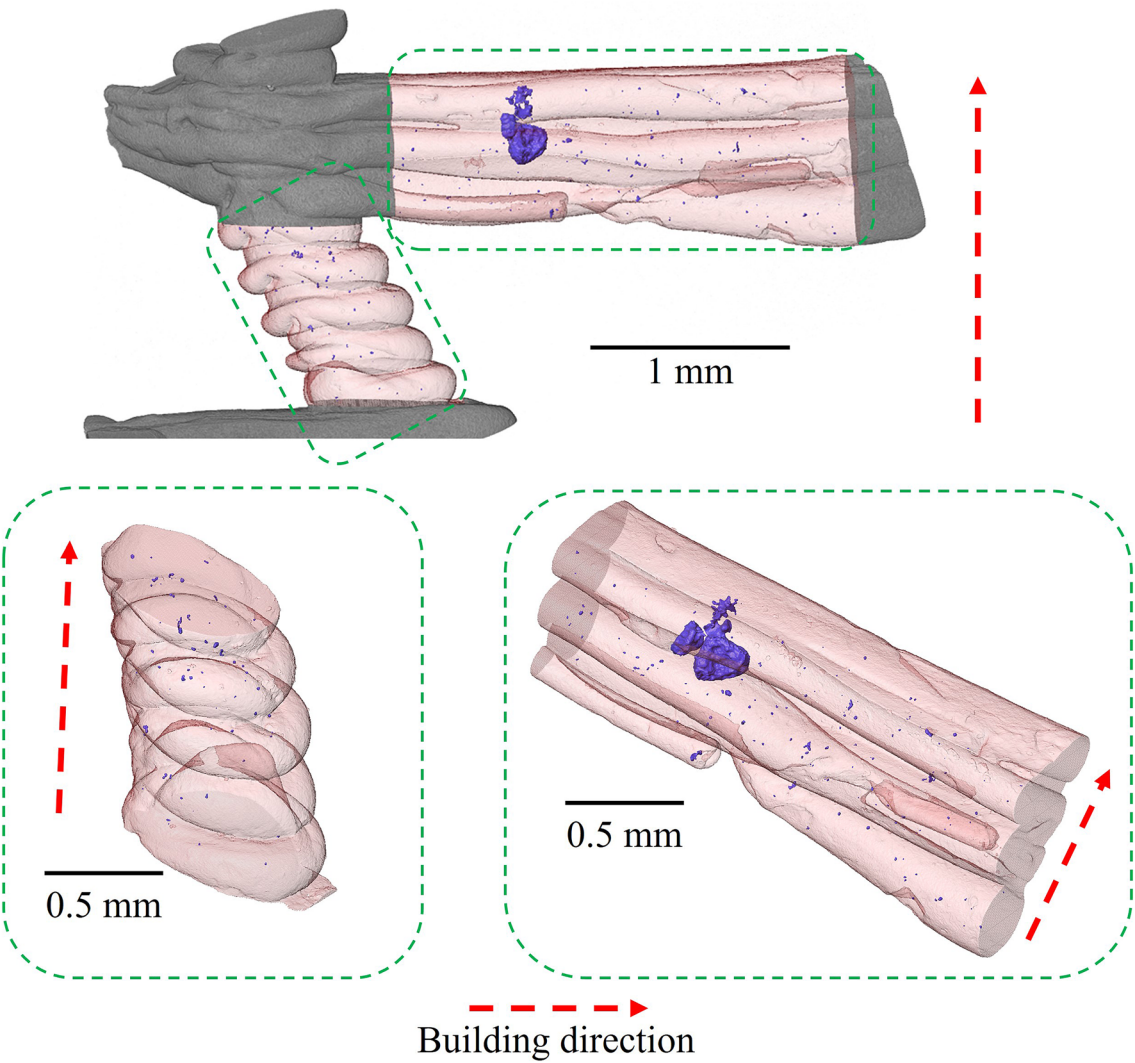
Characteristic defects and internal porosity (rendered purple) observed by µCT (Avizo Standard segmentation) in the rib/struts of periodic cells.

Figures 2 and 3 show the configuration of the rib/struts in the final A356 samples. These highlight the staircase artefacts characteristic of the FFF technique^79^ originating from Step I. The concave effect in the layer boundaries is due to the surface tension on the filament in the rubbery state.

**Figure 4:**
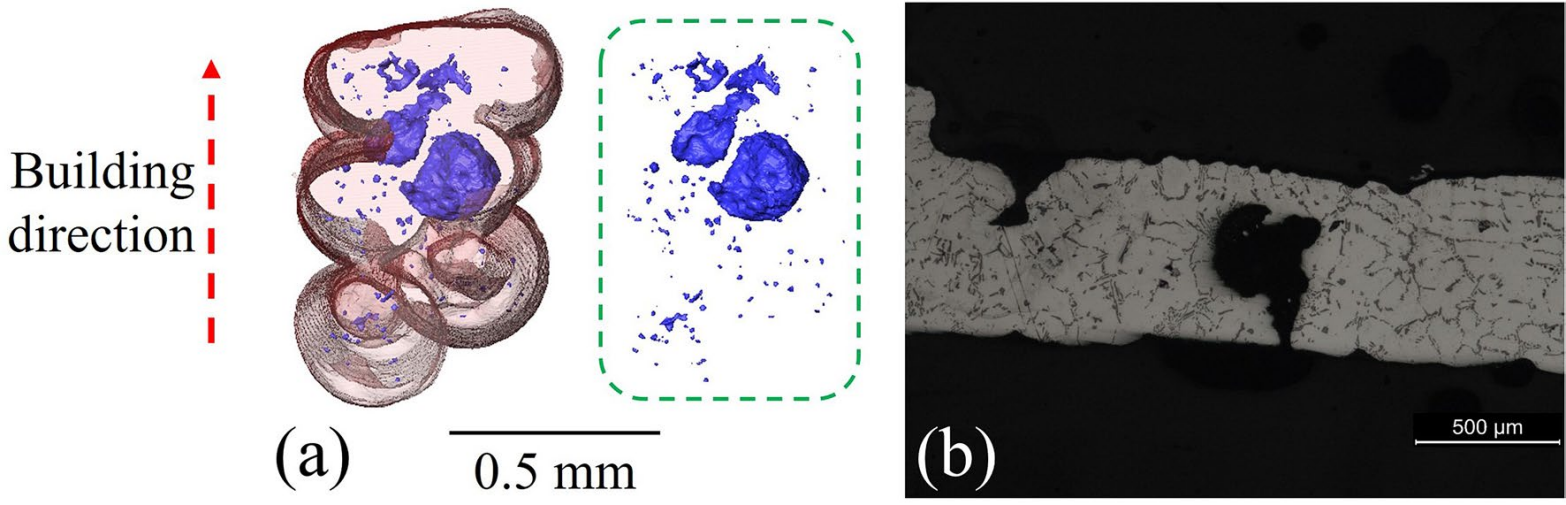
Magnified views of the larger defects found in some horizontal ribs: (**a**) X-ray μCT (Avizo Standard segmentation) and (**b**) optical microscopy.

Figure 3 also shows the distribution of porosity in the rib/struts recorded by μCT. It can be seen that the ribs/struts display fine porosity (~ 3 ± 1 µm, 0.2% volume fraction) that is well distributed and probably originates from the gas already dissolved in the melt. In addition a small number of large (~ 26 ± 15 µm) defects can be observed in the horizontal ribs, as shown in Fig. 3 and detailed in Fig. 4. These defects are promoted by the contact of two alloy melt fronts that meet in horizontal ribs^57^. Although the casting process is performed in vacuum (− 1 bar), due to the high filling rates, residual gas in the casting chamber may become entrained in the mold. This residual gas is not dissolved in the melt and cannot escape due to the low permeability of the plaster mold, and so gets entrapped in the horizontal ribs between two fronts of opposite melt flow. The surface tension and the pressure that is generated by the compression of this entrapped gas is attested to by the quasi-spherical shape of these defects. It is suggested that these defects could be minimized by improving the vacuum. This kind of defect is present in ~ 12% of the horizontal ribs.

The modeling of these defects as spherical inclusions in a cylindrical beam suggests that they generate a 1.6% to 8.6% reduction in the moment of area of the ribs (I_●_ ~ 0.0127 mm^4^ and I_○_ ~ 0.0116–0.0125 mm^4^). It is known that the main deformation mechanism in these cellular lattices is due to strut bending^2,80,81^. Due to the rare occurrence of this defect (12% in horizontal ribs) and the low impact in the moment of area of these ribs, it is not expected that these defects will significantly affect the static mechanical properties. It should be noted however that near-spherical voids may generate stress concentrations in their vicinity and, thus, could influence fatigue properties.

### Microstructural characterization of the cast lattice structures

**Figure 5:**
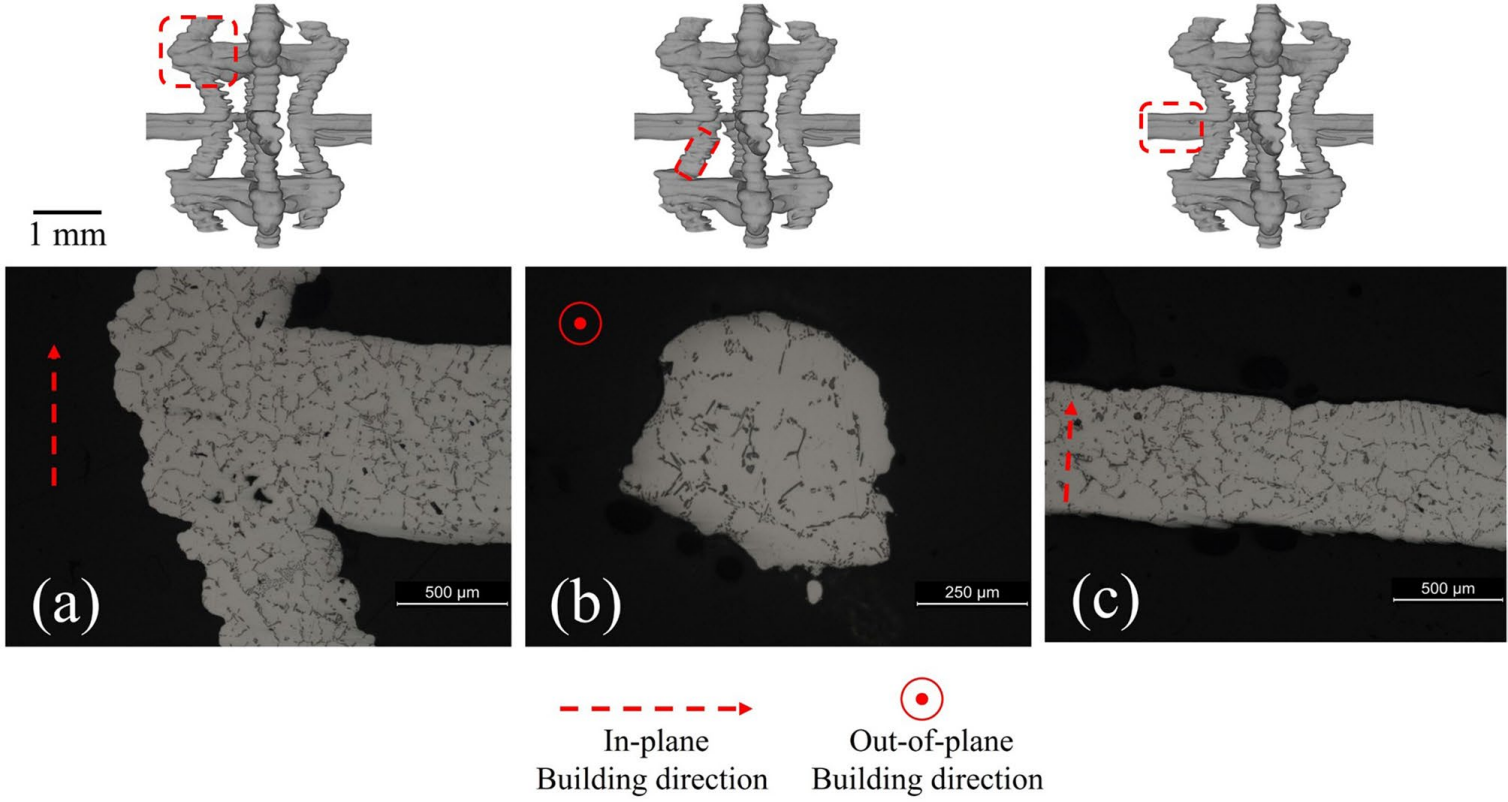
Representative optical micrographs of polished sections for a representative cell showing (**a**) a corner, (**b**) a vertical strut and (**c**) a horizontal rib.

Optical micrographic sections taken from different locations within the structure are shown in Fig. 5, in which the features detailed in 3D in Figs. 2 and 3 may also be observed in 2D. This microstructure exhibits a classical globular/dendritic grain morphology with inter-dendritic second phases characteristic of A356 alloys^58–61^ .

**Figure 6:**
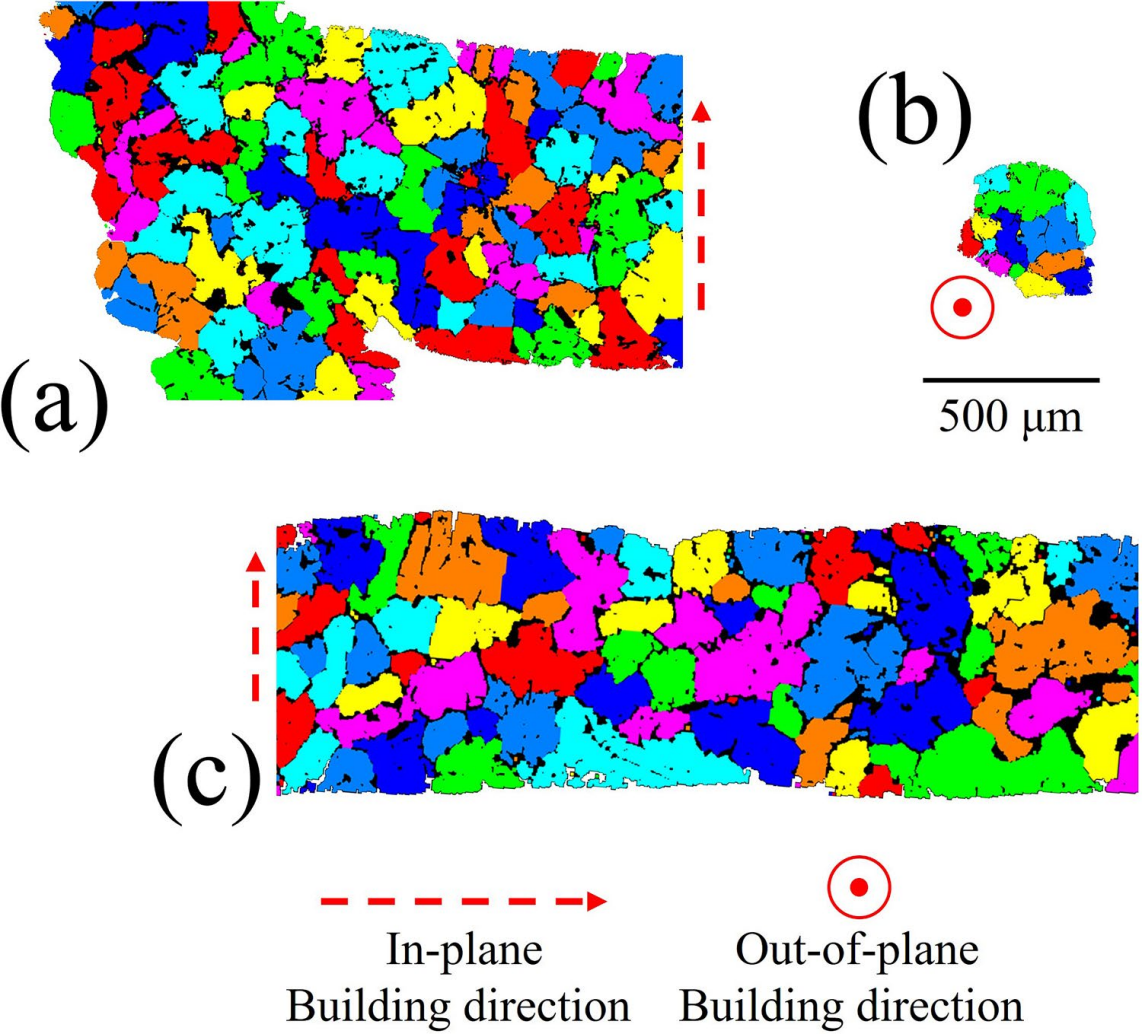
Representative detail of the α-Al grains in the vicinity of (**a**) a cell corner, (**b**) a vertical strut and (**c**) a horizontal rib. The grains were thresholded using Avizo Standard and the random choice of colours used to denote each grain, are not representative of the crystalline orientation of each grain.

**Figure 7:**
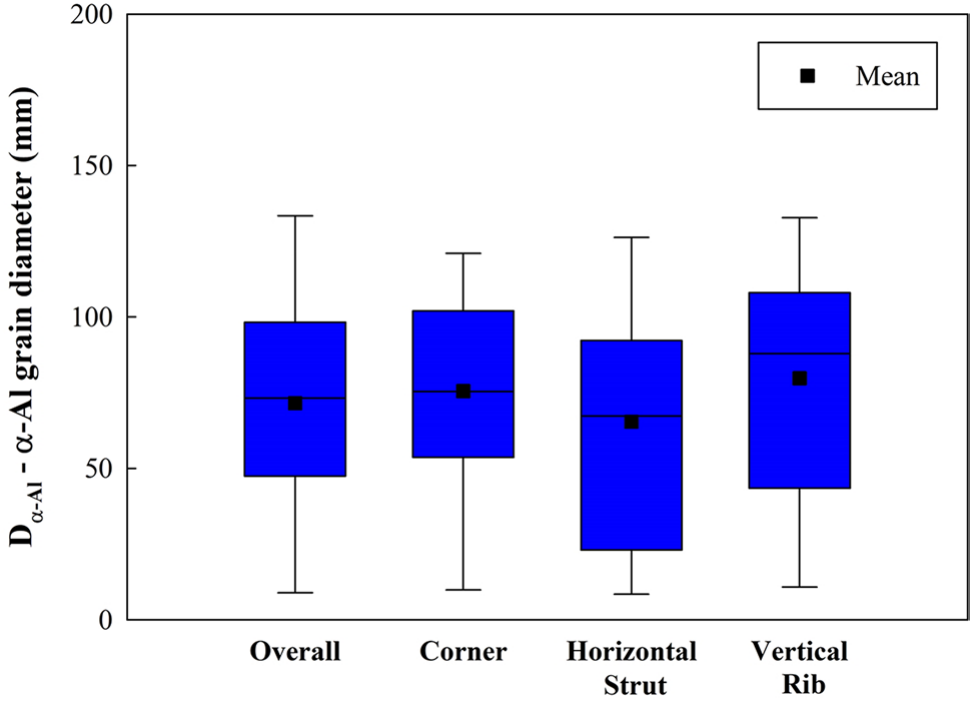
α-Al grain diameter in different fields (n = 50).

Figure 6 illustrates the size and shape/morphology of the α-Al grains characteristic of different locations. From this analysis, it is apparent that the grains have a homogenous size distribution with no apparent anisotropy. Figure 7 which summarises data collected from 27 (regions sampled across 5 manufactured lattices) confirms that there is no significant difference between the α-Al grain diameters (73 ± 39 μm) throughout the lattice structure which compares with (91 ± 46 μm) in the bulk regions of the castings, being recorded as 65 ± 41 μm for the horizontal ribs, 78 ± 46 μm in the vertical struts and 75 ± 46 μm in the corner nodes. Furthermore no significant difference was observed from top to bottom of the lattice structure. This homogeneity in grain size is not surprising given: (i) the rapid filling of the ceramic molds; (ii) the well homogenized temperature of the mold; (iii) the large relative distance to the ceramic mold exterior walls; and (iv) the fineness of the rib and strut configuration. All these aspects promote a uniform cooling rate throughout and hence similar solidification rates across the whole structures.

**Figure 8:**
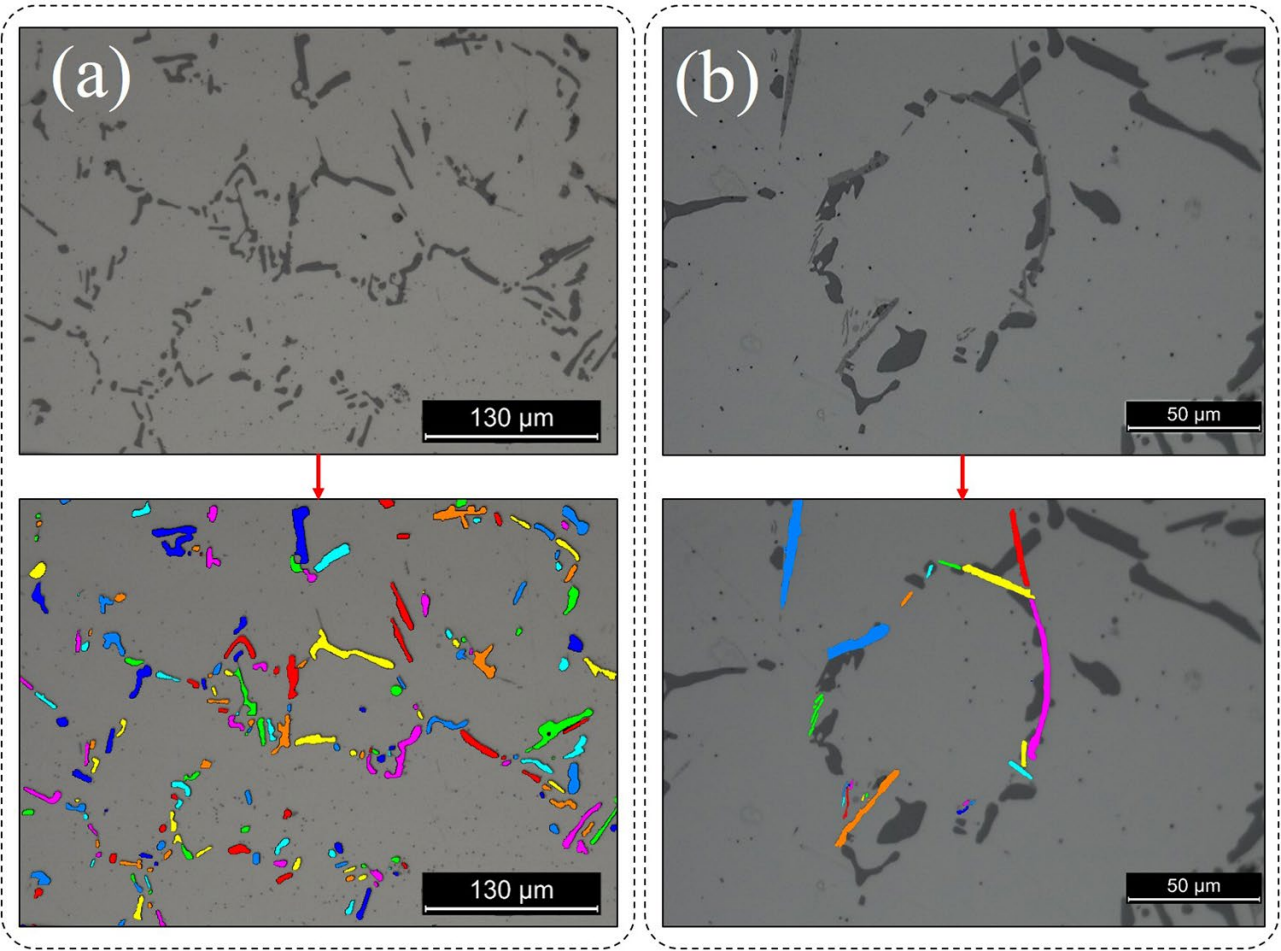
Detail of (**a**) partially spheroidised eutectic Si and (**b**) the needle shaped β-phase intermetallic compounds highlighted in the A356 Al lattice structures for a cell corner (see Fig. 6a). Note the thresholding colors were processed in Avizo Standard and do not portray the orientation of the secondary phases.

The distribution and morphology of the secondary phases are shown in Fig. 8, showing the eutectic Si (Fig. 8a) and the intermetallic compounds (Fig. 8b). It appears that the solution treatment was able to coarsen and spheroidize the eutectic Si by self-diffusion and inter-diffusion^82^, transforming the characteristic fibrous/coral shapes towards a more round configuration after solution treatment^83^. The success of the solution treatment may also be attested by the absence of Mg_2_Si, indicating that it was fully dissolved by the α-Al matrix.

**Figure 9:**
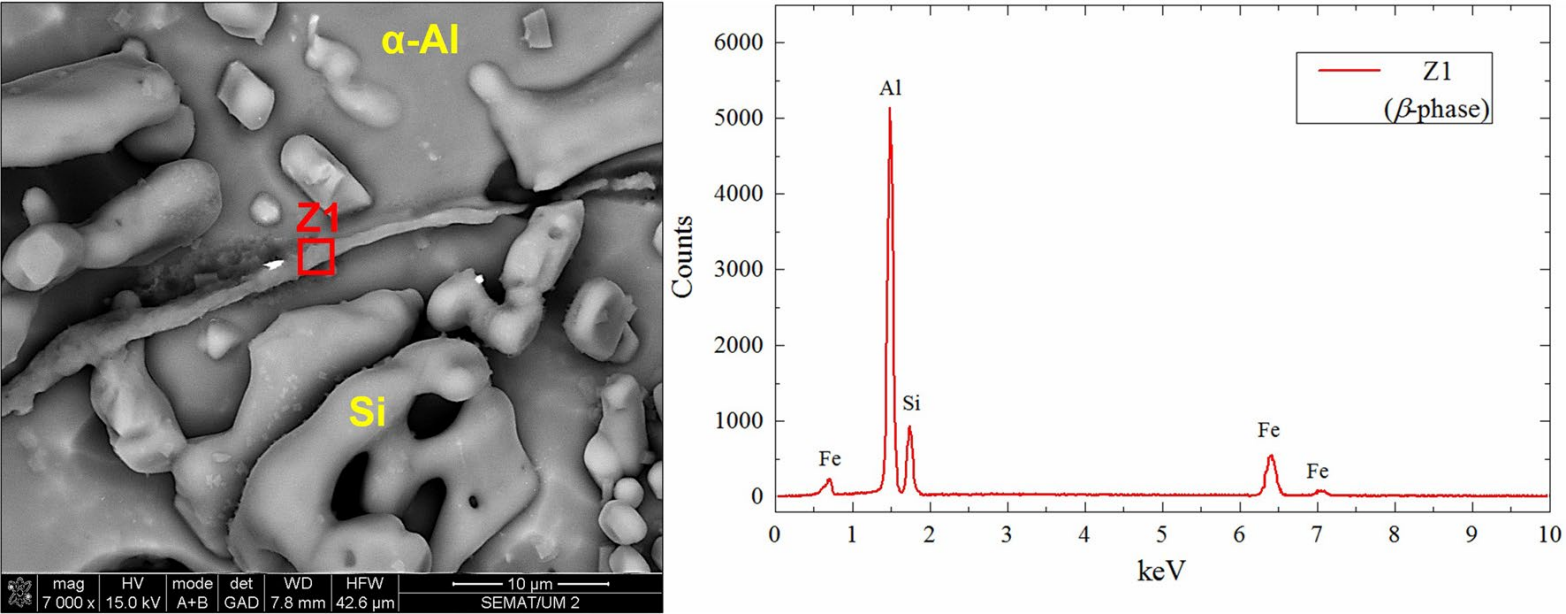
SEM (Secondary electron) image and EDS profile the location of which is indicated by the red box for a deeply etched sample.

In Fig. 8b the β-Al_5_FeSi compound identified as it is the only intermetallic to be needle shaped intermetallic for A356. This was confirmed by scanning electron microscopy (SEM) analysis and energy-dispersive X-ray spectroscopy (EDS, Z1) on deeply-etched samples (Fig. 9). These compounds are promoted by the low solubility of Fe in the α-Al matrix and the presence of Mg^66^. According to these results, it is suggested that the long/thin β-needles form during the casting process, while short/wider β-needles form from solution after quenching and originate from the migration of Mg and Si due to the dissolution of π-Al_8_FeMg_3_Si_6_ compounds^49,84,85^.

## Conclusions

This study describes the characterization of lattice structures formed by a novel fused filament fabrication assisted investment casting technique at the macro-, meso- and microstructural scales. The process involves a two-step protocol, namely the 3D printing of a sacrificial PLA pattern (Step I) that is then used to fabricate the mold for the subsequent casting step (Step II). The following conclusions can be drawn:

1. At the macroscale, statistical analysis shows that the dimensional errors are largely introduced during the first (3D printing) step. Even more accurate lattice structures could be obtained with higher resolution stepper motors and better control of the PLA extrusion process. The second (casting) step is associated with only small volumetric deviations due to phase transitions and shrinkage during solidification;
2. Mesoscale characterization of the intermediate PLA pattern and the final A356 casting shows that staircase defects are introduced by the FFF process. Fine porosity (~ 3 ± 1 µm) is found to homogeneously distributed in the casting in both vertical struts and horizontal ribs due to undissolved gas in the melt. However, larger (~ 26 ± 15 µm) defects may be observed in ~ 12% of horizontal ribs arising from the meeting of two melt fronts. Due to the low permeability of the plaster mold, it is suggested that this defect could be addressed by increasing the vacuum pressure;
3. The microstructure of the cast samples shows grains with both globular/dendritic morphologies. The grain size (73 ± 39 μm) does not significantly change in the different areas of the periodic cells, suggesting that there is no point variation or anisotropy. The subsequent heat treatment is able to refine, agglomerate and spheroidize the eutectic Si. This is proved by the absence of Mg_2_Si and the evidence of β-phase, the only observable intermetallic compound that was originated by π- to β-phase transition;

The dimensional fidelity could be increased further by improvements to the fused filament process for pattern manufacture. More broadly the macro-, meso- and macrostructural evidence suggest that the hybrid additive manufacturing assisted investment casting approach could be applied to other light alloys to fabricate fine periodic lattice structures.

## Materials and methods

### Design and manufacturing of metallic lattice structures by additive manufacturing assisted investment casting

**Figure 10:**
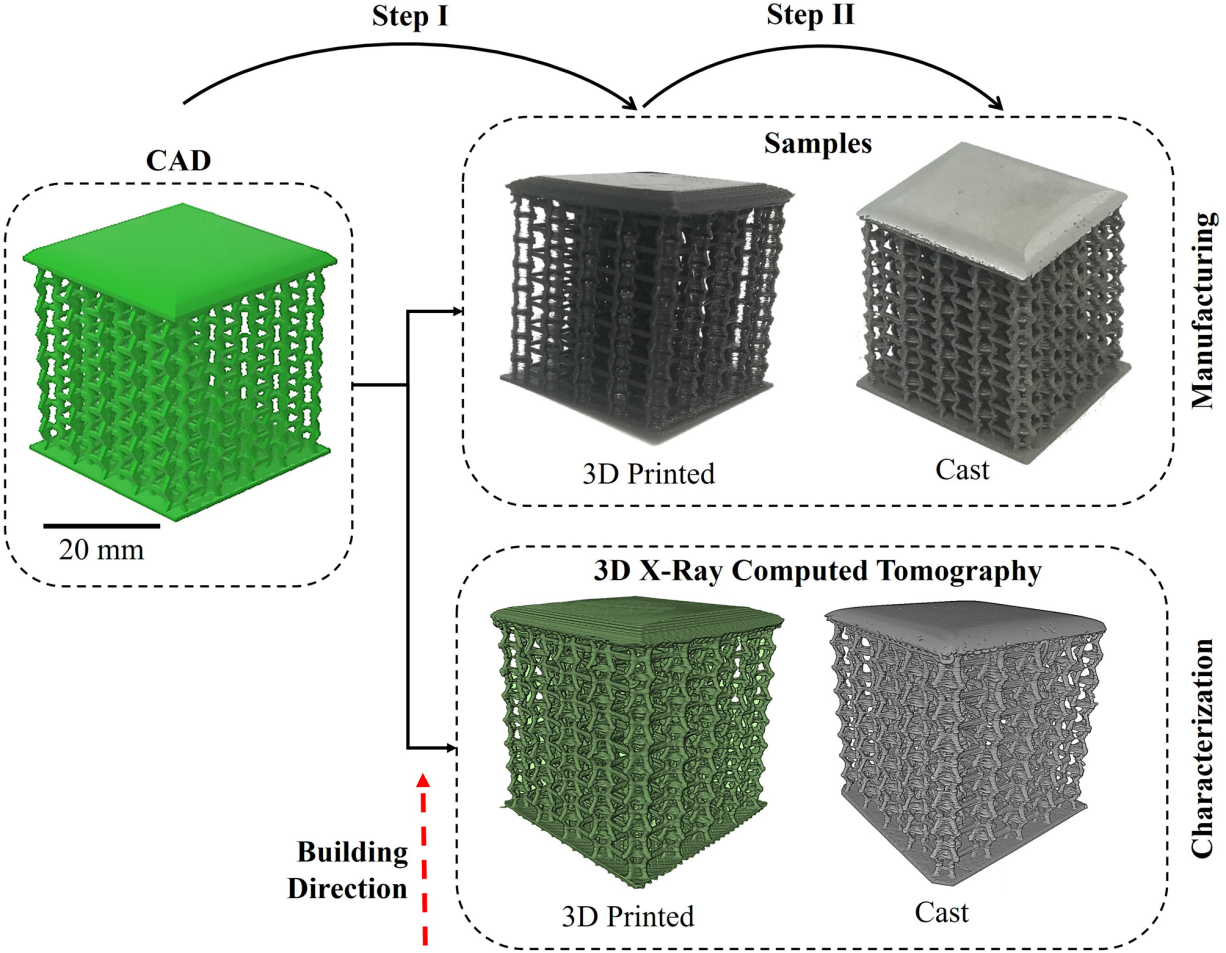
Steps for additive manufacturing and 3D characterization of assisted investment casting of metallic lattice structures from a CAD model to a 3D printed PLA pattern to the as-cast aluminium structure.

A cellular lattice (Fig. 10) was designed by computer added design (CAD) considering a three-dimensional hexagonal ‘honeycomb’ periodic cell, characterized by four horizontal ribs (*h* = 4 mm) and eight oblique struts (*l* = 2 mm) with a circular cross-section (*d* = 0.6 mm). Individual periodic cells were assembled by connecting their common ribs/struts in a 9 × 9x8 array configuration with plates on the top and bottom to enclose the lattice core.

Initially (Step I in Fig. 1) a pattern, representing a replica of the CAD model, is produced from PLA using a FFF technique on a BCN3D Sigma 3D-printer (BCN3D, Barcelona, Spain) using a 0.4 mm nozzle. The extrusion temperature (210 °C), printing speed (10 mm/s) and layer height (0.2 mm) were optimized to assure the successful printing of ten structures without supports.

Step II involves the casting of the samples. The printed patterns from Step I are used to manufacture the casting mold. The PLA patterns are infiltrated with gypsum that is then subjected to a curing cycle (300 °C-3 h, 300–730 °C-5 h, 730 °C-6 h and 730–250 °C-12 h) which hardens the plaster and burns off the enclosed pattern. Meanwhile, A356 ingot was cut into 24 g loads, cleaned and degreased. The alloy was inserted in a SiC crucible with the addition of master alloys (0.05 g Al5Ti1B and 0.07 g Al10Sr) to promote grain refinement and modification of the eutectic Si. After melting in an Indutherm MC15 + induction furnace (Indutherm, Walzbachtal, Germany), the melt was maintained at a temperature of 700 ± 2 °C for 3 min in vacuum (− 1 bar) and magnetic stirring to homogenize the A356 and master alloys. The melt was then cast into the preheated gypsum cylindrical mold (250 ± 5 °C, 80 mm in diameter with a 100 mm length). Upon removing the cast the samples were subjected to a T6-treatment (solution at 540 °C-8 h and artificial ageing at 160ºC-8.5 h)^86^.

### Macro- and meso-structural characterization

The macrostructural characterization of the PLA pattern and final Al lattices was performed by 3D X-ray μCT using a Zeiss Versa XRM-520 μCT scanner (Zeiss, Oberkochen, Germany). Imaging was controlled by an XRM scout-and-scan system (v.1.1.5707.17179; Zeiss, Oberkochen, Germany), using the parameters described in Table 2. This allowed the measurement of actual dimensions/details to assess the deviations that are introduced at each step of the manufacturing process (according to Fig. 1).

**Table 2:** X-ray μCT parameters.

Sample	PLA pattern	A356 sample	
	Macroscale	Macroscale	Mesoscale
Voxel (μm)	28.4	20.9	2.3
Exposure time (s)	6	4	60
Source-sample (mm)	192	130	120
Sample-detector (mm)	36	80	60
Voltage (kV)	100	140	140
Power (W)	9	10	10
Objective	0.39x	0.39x	4x

Mesoscale characterization was also performed by µCT, however, the parameters were adjusted (Table 2) to focus on the ribs/struts of the samples. Such analysis allowed the determination of volume and distribution of porosity/segregation in the final cast sample.

μCT scanning data was reconstructed using the XRM software, while segmentation and 3D visualization was assembled in Avizo Standard (Thermo Fisher Scientific, Waltham, USA). The dimensional measurements of the sample strut/ribs were performed on the original 2D TIFF micrographs using Fiji^87^.

### Microstructural characterization

Microstructural analysis was performed by optical microscopy (Leica DM2500M) to evaluate the microstructural characteristics, morphology of the α-Al grains and secondary phases. Micrographs were taken from 27 cells (in a 9 × 9 matrix configuration) focusing on three distinct regions (cell corners, vertical ribs and horizontal struts) for each analyzed sample, while a total of five samples were analyzed. Fiji software was used to quantify the grain diameter. Measurements were carried out according to ASTM E112 Standard on samples that were previously polished with SiC abrasives down to 11 μm granulometry and finished using 1 μm diamond solution. Avizo Standard was also used to threshold and separate objects in these images to highlight the sizes and shapes of α-Al grains and secondary phases, which were highlighted with different colors.

Deeply etched samples (Keller’s solution—30 s) were observed by SEM (FEI Nova 200) as well as by energy dispersive x-ray spectroscopy (EDS) to characterize and identify the intermetallic compounds (IMCs).

## Data Availability

The datasets generated during and/or analysed during the current study are available from the corresponding author on reasonable request.
